# Mayor Erythropoietic Response after Deferasirox Treatment in a Transfusion-Dependent Anemic Patient with Primary Myelofibrosis

**DOI:** 10.1155/2013/520712

**Published:** 2013-11-06

**Authors:** Del Corso Lisette, Balleari Enrico, Arboscello Eleonora, Ghio Riccardo, Mencoboni Manlio, Racchi Omar

**Affiliations:** ^1^Department of Hematology and Oncology, IRCCS AOU San Martino-IST, Largo Rosanna Benzi 10, 16132 Genoa, Italy; ^2^Department of Oncology, Villa Scassi Hospital, ASL 3, Corso Scassi, 16151 Genoa, Italy

## Abstract

Primary myelofibrosis (PMF) is a myeloproliferative neoplasm frequently complicated by transfusion dependent anemia. Both anemia and transfusion-dependence are associated with a poor outcome, at least in part because of toxic effects of iron overload (IOL). Iron-chelating therapy (ICT) is increasingly used in order to prevent IOL in this setting. Here, we describe the case of a 73-year-old man affected by PMF and severe transfusion-dependent anemia who experienced a dramatic erythroid response after being treated with deferasirox to prevent IOL.

## 1. Introduction

Anemia is a frequent complication of primary myelofibrosis (PMF), either at presentation or during the course of the disease, with an incidence and diagnosis ranging from 50 to 70% [1]. The presence of anemia in PMF is well known to negatively impact survival, and transfusion dependence has been recently reported as a further negative prognostic factor; both of these variables are included in the more used current PMF prognostic scores [2, 3]. 

Although severe anemia could per se indicate a more aggressive disease with higher risk of leukemic transformation, the increased mortality in severely anemic PMF patients does not seem to be related entirely to leukemia but also to the negative effects of chronic low hemoglobin levels on cardiovascular system, and, in the heavily transfused patients, it might also be dependent on the systemic damage of the heart and other organs due to iron overload (IOL). IOL is also believed to increase the infective risk of these already frail patients.

To limit the toxicity of iron excess, iron-chelating therapy (ICT), although not routinely recommended by current guidelines of PMF management, has been recently increasingly proposed in the management of these patients, when transfusion-dependent anemia occurs. A positive effect from ICT on survival in patients with PMF has been already demonstrated by Leitch et al. [4], and it was mainly attributed to a reduction of toxic effects of IOL. A possible direct effect of ICT in improving erythropoiesis of patients with PMF has also been described, even if in a few cases [5–8]. 

Here, we describe a PMF patient with severe transfusion-dependent anemia in which ICT with deferasirox stunningly restored normal hemoglobin levels.

## 2. Case Presentation

A 73-year-old Caucasian otherwise healthy man came to our outpatient's clinic in August 2011 because of neutrophil leukocytosis and splenomegaly. Blood counts were as follows: white blood cells (WBC) 28.2 × 10^9^/L, hemoglobin (Hb) 11.5 g/dL, and platelets (Plt) 350 × 10^9^/L. Physical examination was unremarkable with the exception of mild splenomegaly (lower margin 5 cm under costal margin). Folic acid and B12 vitamin serum concentrations were within normal ranges.

Examination of a peripheral blood smear revealed the presence of marked anysopoichilocytosis with several dacriocytes and orthochromatic erythroblasts, together with immature myeloid precursors (myelocytes and metamyelocytes) and 1% of myeloid blasts.

A bone marrow trephine biopsy showed a typical “myeloproliferative” pattern with myeloid hyperplasia and decreased erythropoiesis together with clusters of abnormal megakaryocytes; a grade I fibrosis (reticulin fibrosis according to 2008 World Health Organization (WHO) criteria [9]) was also observed. The assessments of Bcr/Abl rearrangement and JAK2 V617F mutation were both negative. According to 2008 WHO criteria [9], a diagnosis of PMF was therefore made, with an International Prognostic Score System (IPSS) [10] score of 2 (intermediate 2 risk) and a Dynamic International Prognostic Score System (DIPSS) [11] score again of 2 (intermediate 1 risk). 

After few months of clinical observation, the patient progressively developed extreme leukocytosis (WBC 100 × 10^9^/L), mild thrombocytopenia (Plt 120 × 10^9^/L), and worsening of the normocytic anemia (Hb 10.0 g/dL). Splenomegaly progressively increased, with recurrent abdominal discomfort. 

In December 2011, the DIPSS of the patient had increased to a score of 5 (high-risk), and a cytoreductive therapy with hydroxyurea and low-dose prednisone was consequently started, with an only partial response in WBC counts; because of worsening of anemia, treatment with erythropoietin-alpha (EPO) (40,000 U/week) was started in May 2012, with no improvement of anemia, which actually rapidly further worsened; in June 2012, Hb decreased to a nadir of 5.9 g/dL, and the patient became transfusion dependent; transfusion's requirement rapidly increased to 4–6 packed red blood cells (PRBC) units per month. Splenomegaly and abdominal pain kept worsening, with the spleen reaching 30 cm in size, as measured by US scan in its longitudinal axis. The patient refused treatment with thalidomide, and in July 2012 splenectomy was performed. After splenectomy, the patient developed thrombocytosis (Plt 650 × 10^9^/L), with a transient (few weeks) improvement of anemia, but thereafter he remained transfusion dependent with a mean monthly requirement of 5 PRBC units. Hepatomegaly was not observed. Treatment with hydroxyurea and EPO was maintained.

Because of the appearance of IOL, as indicated by a ferritin level of 1424 *μ*g/L with transferrin saturation of 87%, after a total of 35 PRBC units transfused, in January 2013 the patient received ICT with deferasirox. Because of the presence of a mild impairment of renal function (creatinine clearance < 60 mL/m), a dose of 10 mg/Kg daily was used instead of the usual dose of 30 mg/Kg/day. After 4 weeks of treatment, we were surprised to observe a significant increase of Hb (9.1 g/dL) with no further transfusional need. After 8 weeks of deferasirox therapy, Hb raised up to 13.3 g/dL and EPO was stopped. A steady platelet's decrease was also observed, which returned within normal ranges, while leukocytes counts remained elevated (mean WBC 70 × 10^9^/L). After only two months of ICT, ferritin level decreased to 223 *μ*g/L and deferasirox was stopped. Because of an increase of ferritin up to 800 *μ*g/L, ICT was reintroduced at the dose of 5 mg/Kg daily in April 2013, and it is ongoing at the time of present writing. ICT has been well tolerated with only self-limiting G1 diarrhea, and the patient, after 6 months from the beginning of deferasirox, is in good clinical condition with stable Hb around 14.0 g/dL, thus experiencing a full anemia response according to the recently revised IWG-MRT and ELN response criteria for PMF [12].

**Figure 1:**
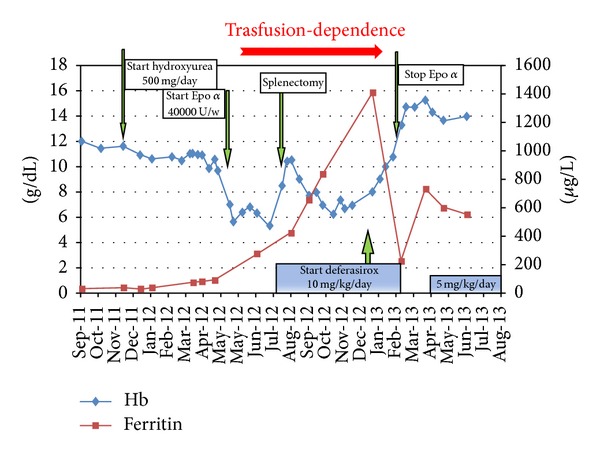
Hemoglobin and ferritin variations during the clinical course.

Figure 1 shows the evolution of hematological parameters in relationship with the various treatments.

## 3. Discussion

PMF is a myeloproliferative neoplasm frequently complicated by transfusion-dependent anemia. With the exception of few cases eligible to bone marrow transplantation, no known treatment is able to alter the natural course of the disease. Given the detrimental effects of anemia and of IOL due to a prolonged transfusional support, any treatment able to improve anemia and transfusion-dependence could have a significant impact on patients' quality of life and life expectancy. The impact of IOL on survival of PMF patients is actually controversial; two different studies of the same institution on this topic have indeed given different results [13, 14]. Nevertheless, an increasing amount of evidence seems to indicate that IOL has a detrimental effect on clinical course of PMF patients and that ICT might overcome it. 

In fact, because many transfused PMF patients may experience a relatively long survival, increasing interest has focused on IOL prevention by adequate ICT. According to the more commonly used prognostic scores, a patient affected by PMF with a high-risk score, as was the case here described at the time of starting ICT, has indeed a median survival of 27 and 18 months, respectively, for IPSS and DIPSS. Furthermore, one retrospective study in PMF showed a positive effect of ICT on survival, postulating a decreased toxic effect from IOL with the consequent reduction of both cardiac and infectious mortality [4]. In the case here described, an unexpected hemoglobin increase was observed after the start of ICT. Although this erythroid response might be stochastic and unrelated to iron chelation or it could also be dependent on a delayed response to splenectomy [15], the strict relationship between deferasirox treatment and hemoglobin improvement observed in our patient is impressive and suggests a direct role of ICT in improving erythropoiesis of this patient. A bone marrow reassessment was not performed in our patient either at the progression or after improvement following the start of ICT, thus precluding any evaluation of possible clonal evolution of the disease, but the relative stability of WBC and Plt counts suggests that this was not the case. 

Several emerging lines of evidence actually indicate that ICT can improve hematopoiesis and lead to a reduction or abolition of transfusion dependence in PMF, as we observed in our patient [4–8]. These data are very sparse and mainly derived from single case descriptions, but they are suggestive of a real biological phenomenon. A similar positive impact on transfusion dependence has been also described in patients with myelodysplastic syndromes (MDS) [16–19] thus suggesting the absence of a specific correlation between hematopoietic improvement due to ICT and the type of disease. 

Erythroid responses to ICT were varying defined as reduction in transfusion requirement or as an increase in hemoglobin levels but all concluded for a positive response that in a post hoc analysis of MDS patients from the EPIC study showed a significant improvement of erythropoiesis after ICT with deferasirox in 21.5% of cases [17]. 

Several possible mechanisms by which ICT can improve erythropoiesis have been proposed: a direct cytoreductive effect of ICT on the neoplastic clone was firstly suggested by Jensen [19], while reduction of oxidative species—which are believed to correlate with inefficient erythropoiesis [20, 21]—or inhibition of NF-*κβ* leading to a reduced transcription of antiapoptotic factors [22] has been more recently proposed.

The case here described showed an impressively strong positive impact of ICT in erythropoiesis of our patient, who experienced a complete and durable (six months at the time of present writing) resolving of a severe transfusion-dependent anemia. Further prospective and larger studies are necessary in order to confirm the exact role of ICT with deferasirox in the improvement of erythropoiesis of patients with PMF and to clarify the mechanism(s) underlining this phenomenon.
